# Physical rehabilitation financing in Iran: a policy analysis using Kingdon’s multiple streams

**DOI:** 10.1186/s12913-021-06447-8

**Published:** 2021-05-03

**Authors:** Saeed Shahabi, Parviz Mojgani, Hosein Shabaninejad, Ahmad Ahmadi Teymourlouy, Masoud Behzadifar, Kamran Bagheri Lankarani

**Affiliations:** 1 Health Policy Research Center, Institute of Health, Shiraz University of Medical Sciences, Shiraz, Iran; 2 Iran-Helal Institute of Applied Science and Technology, Tehran, Iran; 3 Research Center for Emergency and Disaster Resilience, Red Crescent Society of The Islamic Republic of Iran, Tehran, Iran; 4 Population Health Sciences Institute, Newcastle University, Newcastle, UK; 5 Department of Health Services Management, School of Health Management and Information Sciences, Iran University of Medical Sciences, Tehran, Iran; 6 Social Determinants of Health Research Center, Lorestan University of Medical Sciences, Khorramabad, Iran

**Keywords:** Physical rehabilitation, Rehabilitation, Financing, Health policy, Kingdon’s multiple streams

## Abstract

**Background:**

Adequate financing is a crucial function, securing that physical rehabilitation services (i.e., physiotherapy, occupational therapy, prosthetics and orthotics) are available with no financial hardship. Like many other countries, despite the adoption of various policies and strategies in recent decades, Iran enjoys no desirable physical rehabilitation financing (PRF). Accordingly, this qualitative study aimed to explore the PRF-related strategies and issues as well as their impacts on relevant policies in Iran.

**Methods:**

An analysis of PRF-related policies was conducted in Iran using semi-structured interviews and policy documents review. Purposive and snowball sampling techniques were employed to select key informants, including health-policy makers, civil society, rehabilitation-policy makers, university professors, and practitioners. Thematic analysis was used to analyze the collected data. The analysis was framed within Kingdon’s multiple streams.

**Results:**

The hindering factors for desirable financing were weak insurance coverage, lack of sustainable financial resources, fragmented financing, lack of split between provider and financer, high-cost of physical rehabilitation services, low engagement of relevant experts in policy-making processes, and corrupt activities. In the policy stream, the following factors were highlighted: involvement of sustainable financial resources, the use of external revenue sources, allocated resources’ earmarking, the integration of the current funds to have better pooling, the use of incentive and timely payment mechanisms, the implementation of strategic purchasing principals, and the employment of effective rationing strategies. Moreover, parliament support, changes in administrations, international effects, pressures from interest campaigns and NGOs, and international sanctions were found as factors affecting the politics stream.

**Conclusion:**

The study findings revealed that a variety of national and international factors affect PRF-related issues in Iran. The recently enacted laws indicate that the PRF policies have already been on the national health political agenda. The study reflected the multifaceted nature of barriers to optimal PRF in Iran.

## Introduction

Rehabilitation, as a part of the Universal Health Coverage (UHC) [1], which is targeted by the Sustainable Development Goals (SDGs) 3 [2], needs to be strengthened to provide high-quality and affordable services to the ones in need. However, progress towards universal rehabilitation coverage has not been desirable, particularly in low- and middle-income countries [3]. In other words, there is a significant gap between the demand for rehabilitation services and their supply [4]. Due to the rapid growth of the elderly population, an increase in non-communicable diseases (NCDs), and an increase in traffic accidents and natural disasters, rehabilitation has been introduced as the twenty-first century treatment strategy [5]. In response, the World Health Organization (WHO) has launched several initiatives and guidelines to improve the rehabilitation services in the health-care sector, such as Rehabilitation in Health Systems [6] and Rehabilitation 2030: A Call for Action [7].

According to the evidence, adequate financing is a crucial function to ensure that physical rehabilitation services (PRS) (among others, physiotherapy, occupational therapy, prosthetics and orthotics) are available with no financial hardship [6, 8]. Financing is one of the four main functions of the health system, which has been considered a control knob [9]. Health financing includes four arrangements: (1) raising revenues, (2) pooling funds, (3) purchasing services, and (4) designing benefits [10]. Despite the significance of financing in the provision of health services such as PRS, in almost all countries there is no specific financial resource for PRS [7, 11]; hence, many of these services are with no insurance coverage and are mainly provided by the private sector [4]. On the other hand, since people with disability and vulnerable groups form a major proportion of the service recipients, they face catastrophic health expenditures (CHEs) [12], and this is against the goals of UHC [13].

Like many other countries, no desirable physical rehabilitation financing (PRF) exists in Iran, despite the adoption of various policies and strategies in recent decades [14, 15]. Consequently, out-of-pocket (OOP) payments are the most common and predominant PRF mechanism in Iran [16]. According to some recent studies, financial issues are highlighted as one of the main barriers to the reception of rehabilitation services in Iran [14]. Substantial OOP payments have adverse effects on health-care equity [17, 18]. In this regard, due to the high prevalence of NCDs such as neurological diseases (e.g. stroke, multiple sclerosis, and cerebral palsy) [19] and musculoskeletal disorders (e.g. low-back pain, neck pain, rheumatoid arthritis, and osteoarthritis) [20], and also population aging (which is expected to make up a quarter of the Iranian population in coming years) [21], the provision of appropriate and affordable PRS is needed and desirable. This makes the PRF issues be put on the political agenda. Since policies play an important role in health issues, it is critical to identify and understand essential factors affecting the agenda-setting process [22].

This qualitative study aimed to explore the PRF-related strategies and issues as well as their impacts on relevant policies in Iran. Further, the research team was to investigate how policies were evolved in this context and why they were not conducted as expected to prepare a favorable platform for adopting effective policies. To this end, Kingdon’s multiple streams theory [23] was considered in this study.

## Methods

### Study setting

The present study was conducted in the Islamic Republic of Iran, as the second most populous country in the Middle East region. The following institutions and stakeholders are involved in financing of PRS in Iran: Social Security Organization (SSO), State Welfare Organization, the Ministry of Health and Medical Education (MHME), Iran Health Insurance Organization (IHIO), Planning and Budget Organization, the Parliament, Social Security Office of the Armed Forces, Foundation of Martyrs and Veterans Affairs, Iranian Red Crescent Society, government, and private supplementary insurance companies [16, 24]. Given that a limited portion of PRS is covered by insurance (especially supplementary insurers), the coverage of costs is very low for all individuals, except for special groups such as veterans [25]. Furthermore, the adoption of privatization and outsourcing policies in the governmental rehabilitation units has provided a majority of services by the private sector, which usually have higher tariff rates [16]. Accordingly, OOP is a common form of payment, and impoverishing expenditures [1] and CHEs [14] are imposed on many households. In response, during the last decades, Iran’s government and parliament have developed several policies to improve financing and providing PRS (e.g. Principles 29 and 43 of the Constitution, Universal Health Insurance Law (1994), Comprehensive Law on the Protection of the Rights of Persons with Disabilities (2004), and Law on the Protection of the Rights of Persons with Disabilities (2018)), especially those needed by individuals with disabilities; however, no significant improvement is made in practice [26, 27].

### Study design

This research was a qualitative study evaluating the policies and issues regarding the PRS financing in Iran using comprehensive document review and in-depth semi-structured interviews.

### Document review

In the first place, we conducted a comprehensive search to find policy documents related to PRF policies and issues in Iran. Our review included both scholarly publications and grey literature. First, PubMed, Scopus, Embase, Web of Science, Physiotherapy Evidence Database (PEDro library), Rehab Data, and ProQuest databases were searched from inception until June 2019. Search strings were initially formulated for PubMed and then adapted to be employed in the other databases. Additionally, Google Scholar and OpenGrey as well as key journals (Iranian Rehabilitation Journal, Archives of Rehabilitation, International Journal of Health Policy and Management, etc.) were searched manually to detect the relevant grey literature. Second, the official websites of the relevant organizations and stakeholders, including the State Welfare Organization, the Islamic Parliament Research Center, MHME, SSO, IHIO, Ministry of Cooperatives Labor and Social Welfare, Social Security Office of the Armed Forces, Foundation of Martyrs and Veterans Affairs, Iranian Red Crescent Society, Scientific Associations (Physiotherapy, Occupational therapy, prosthetics and orthotics), and Eastern Mediterranean Region Office (EMRO), were appraised to explore relevant national, regional, and international laws, policies, protocols, reports, and guidelines. The following keywords were considered throughout the search procedure: (finance OR financing OR fund OR funding OR insurance OR “revenue collection” OR pooling OR purchasing OR “benefit package”) AND (policy OR policies OR programs OR strategies OR plans OR solutions) AND (rehabilitation OR “rehabilitative services” OR physiotherapy OR “physical therapy” OR “occupational therapy” OR “ergo therapy” OR orthosis OR orthoses OR orthotics OR prosthesis OR prostheses OR prosthetics) AND Iran.

### Semi-structured interviews

In-depth semi-structured interviews were conducted via phone calls, internet, and face-to-face meeting from July to November 2019. Purposive and snowball sampling techniques were employed to select the key Iranian informants, including health-policy makers, civil society, rehabilitation-policy makers, university professors, and practitioners. Due to the familiarity of the research team members with physical rehabilitation field and also health financing system in Iran, they had enough knowledge about the potential participants. Therefore, purposive sampling was used first and in later stages we tried to find other suitable samples by snowball sampling. The recruiting procedure continued until data saturation was achieved. In fact, the last two interviews, which had duplicate data and no new information was found during them, were considered to confirm data saturation. The participants were contacted via e-mail and an instant messaging application, and the date, time, and method of the interviews were determined. Before each interview, written informed consent forms were signed after explaining the objectives of the study and general information about interviewer. The forms were then collected from the participants. Furthermore, individuals were guaranteed to be anonymous throughout the research, and they were free to leave the study at any stage, if they wished.

All the interviews were performed in a quiet room without any third person by the first author (a male PhD candidate with a rehabilitation background) with regard to an interview guide developed in accordance with Kingdon’s multiple streams theory (Table 1). The interview sessions were recorded digitally using two audio recorders, and the interviewer took handwritten notes conscientiously during the interviews. All the anonymously recorded files were transcribed verbatim by S.SH. To remove any potential risk of bias, there was critical reflexivity throughout the data collection and analysis phases. Furthermore, the interviewer described his scientific interests and executive background during the interviews. Moreover, with the aim of promoting the rigor and trustworthiness, the following methods were considered to ensure the confirmability, dependability, transferability, credibility and authenticity criteria (1) Member-checking by co-authors and participants (confirmability); (2) engaging three authors in the analysis process (dependability); (3) using purposive sampling (transferability); (4) peer debriefing, data and method triangulation, as well as long-term immersion of the first author in this field (credibility), and (5) inserting citations from various participants (authenticity).

**Table 1:** Interview guide

Questions	
1. Problem stream: what issues and challenges make the financing of physical rehabilitation services a problem in Iran?	
2. Policy stream: what potential policies and solutions have been developed and adopted by stakeholders to address the financing of physical rehabilitation services in Iran?	
3. Politics stream: what are the political factors and issues that can affect the policies related to financing of physical rehabilitation services in Iran?	

### Conceptual framework of the study

**Fig. 1:**
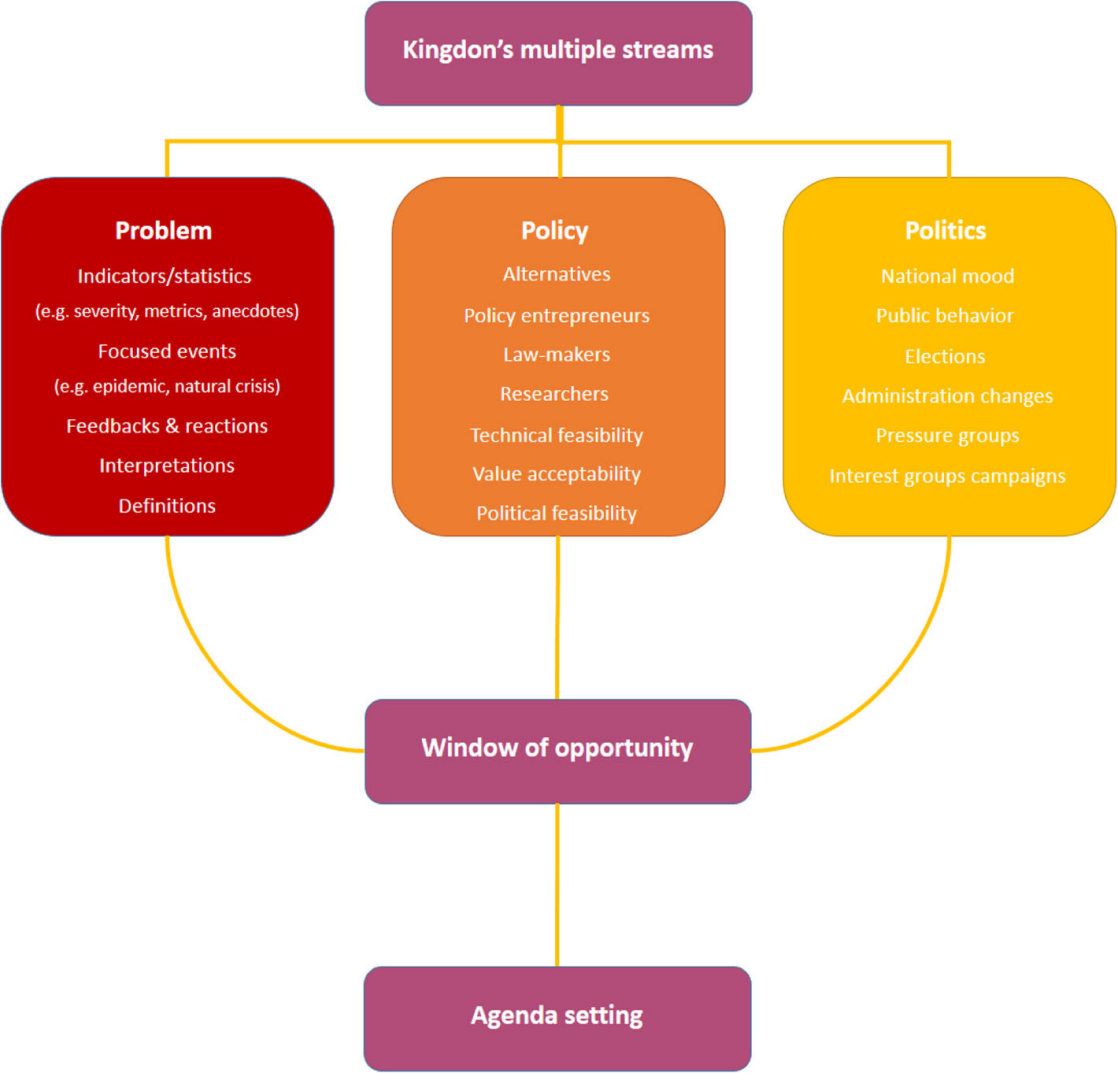
Diagrammatical representation of the Kingdon’s multiple streams

Kingdon’s multiple streams theory was employed to evaluate the PRF-related policies in Iran (Fig. 1). This framework is one of the most commonly used conceptual tools used for the analysis of different policies, including health-related policies [28]. According to this framework, multiple streams (or events) must collide to open up the “window of opportunities” for an interested issue and be considered by decision- and policy-makers during agenda-setting process [29]. Then policy entrepreneurs take an advantage from these opportunities to propagate and progress the policies. These three streams include politics, policy, and problem [30].

The politics stream describes the national mood and behavior, shifts in the governing political parties, changes in government administrations and agencies, turnover, pressures, and interest groups campaigns [31]. The policy stream also encompasses a numbers of actors, including political parties, interest groups, policy entrepreneurs, law-makers, researchers, and professionals, who can address a variety of issues and problems to affect the agenda-setting process. In general, these actors spare their efforts to provide and develop the potential initiatives and alternatives [31]. To survive, the proposed initiative must have the following specific criteria: ability to anticipate future constraints (the possibility of initiatives with public, financial, and political acceptability), technical feasibility, and value acceptability. Another stream of this framework is the problem stream and includes three mechanisms of indicators, feedback mechanisms on the current policies, and focused events [32]. The problem may be defined with regard to individuals’ values in a particular situation or in comparison and benchmarking with other regions and countries. This stream is recognized when an issue becomes socially important, and the government feels the necessity of adopting some measures [33].

Indeed, given the nature of the rehabilitation sector in Iran and the participation of various stakeholders and actors in the development and adaptation of related policies, these three streams can be very helpful in recognizing PRF issues.

### Analytical approach

Thematic analysis was used to analyze the collected data [34]. Data analysis was conducted with data collection simultaneously. In this study, Braun and Clarke’s methodology was adopted, which consisted of six phases (namely getting familiar with data, establishing initial codes, discovering, evaluating, labeling emerged themes, and preparing a final report) [35]. This approach is considered appropriate for informing policy formulation and development [34]. In other words, it allows researchers to explore the anticipated and unanticipated themes. Four authors (S.SH, P. M, A. A, and H.SH) participated in the analysis procedure and read the written texts frequently. They open-coded the data. Then the conceptual framework was considered to refine the identified codes and detect the relationships among the emerged themes. Discussion and consensus approaches were used to solve any disagreements between the authors. Data analysis was performed manually, and MAXQDA 11 software (VERBI GmbH Berlin, Germany) was used if required.

#### Ethics

The Ethics Review Committee of Iran University of Medical Sciences (IUMS), Tehran, Iran (IR/IUMS/REC/1397/889), provided the ethical approval for this study. Moreover, a signed written informed consent form was received from all the participants.

## Results

**Fig. 2:**
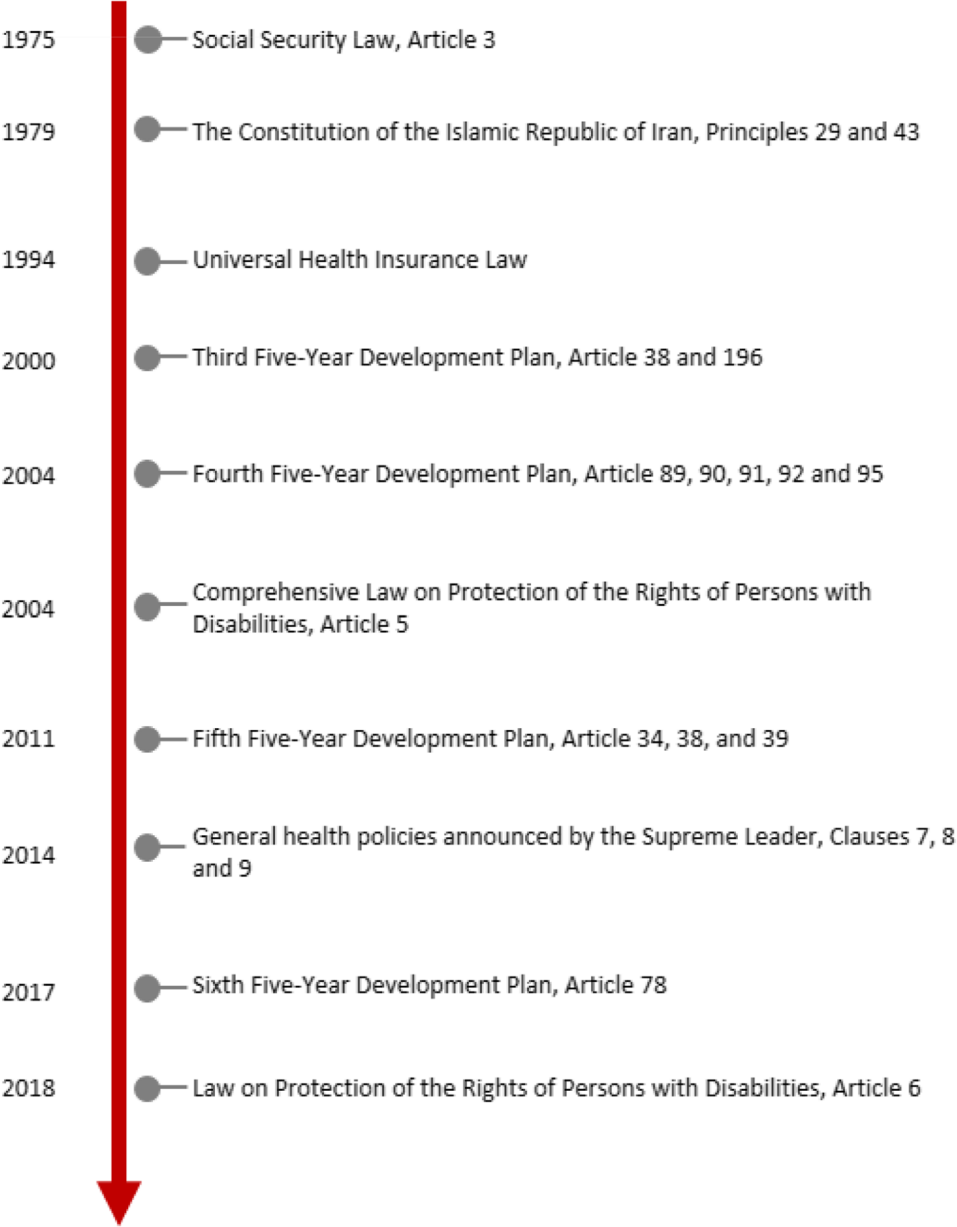
History of key policy documents

In total, 39 interviewees, including policy-makers, academics, practitioners, and civil society, agreed to participate in this study (Table 2). Furthermore, several policy documents were found, which were directly or indirectly related to PRF (Fig. 2). The findings are demonstrated below in accordance with Kingdon’s multiple streams (i.e., problem, policy, and politics).

**Table 2:** Characteristics of participants

Participants	No.
Health policy-maker	6
Rehabilitation policy-maker	4
Physiotherapist	6
Orthotist	5
Prosthetist	3
Occupational therapist	4
Faculty member	8
Civil society	3

### Problem stream

#### Weak insurance coverage

Data analysis showed that only a limited portion of PRS are covered by health insurance in Iran. In addition, they cover a small part of the costs of services, and the payment system is highly time-consuming. In general, only limited sessions of routine physiotherapy interventions are covered by health insurance and SSO as basic insurances, and other services or more sessions should be covered by private and supplementary insurers. Accordingly, a major share of the costs is paid by patients and households directly, and this issue in certain cases would lead to impoverishment and catastrophic expenditures.

#### Lack of sustainable financial resources

The respondents stated that the dependence of the health and welfare sector’s budget on the revenues of raw oil, gas and other unsustainable resources has posed a serious challenge to health system financing, including rehabilitation services. In addition, there are often no such revenues due to sanctions and international relations; therefore, policy-makers emphasized the movement towards sustainable funding sources.

#### Fragmented financing

The existence of multiple health insurance funds in Iran has prevented the proper pool of revenues and effective redistribution mechanisms to reduce the financial burden and promote equity. The participants stressed that the lack of cross-subsidized approaches has made some individuals, especially low-income and vulnerable groups, take fewer insurance benefits. This can be regarded as an example of cream-skimming in the insurance industry.

#### Lack of provider-financing split

A majority of the participants believed that there is no competition to improve contracting and strategic purchasing approaches since many Iranian health insurance companies have their own health facilities (such as clinics and hospitals). On the other hand, such a structure has made insurers reluctant to buy services from other sectors such as non-governmental organizations (NGOs) and private centers.

#### High-cost of physical rehabilitation services

The participants, especially policy-makers, noted that insurers fail to cover all costs since the PRS costs are extremely high. Further, recent inflation rates have made these services even more expensive.

#### Lack of sufficient awareness among decision- and policy-makers

A number of university professors argued that senior policy-makers are not fully aware of rehabilitation services and are more likely to view them as luxury services. Accordingly, these services are considered a series of expensive services not being worth insurance coverage.

#### Low participation of relevant experts in policy-making processes

Lack of participation of rehabilitation specialists, as well as other stakeholders such as professional associations and relevant NGOs, in policy-making processes, was introduced as a major obstacle in the financing process of rehabilitation services in Iran. In other words, the participants argued that many relevant policies are developed and adopted by groups not familiar with the field of rehabilitation.

#### Corrupt activities

More importantly, many participants pointed to the corruption existing in the PRF process, as in other sectors of the health system. One policy-maker believed that some service providers offer fake invoices to insurers. On the other hand, practitioners criticized policy-makers, arguing that since the physicians and not the therapists are mostly involved in the policy-making process, they put their services in insurance benefit packages and ignore rehabilitation services.

#### Lack of comprehensive health technology assessment

Another challenge of PRF was the lack of a comprehensive health technology assessment, as mentioned in many interviews. In other words, the lack of rehabilitation intervention evaluation in terms of clinical effects and economic benefits has made many participants view them as third-level services not to be included in basic health packages.

#### Significant prevalence of non-communicable diseases

Notably, the incidence and prevalence of NCDs are increasing in Iran, as in many other countries. Given the increasing rate of aging in Iran and the increasing frequency of disabilities from traffic accidents, musculoskeletal disorders, and neurological diseases, individuals’ access to affordable rehabilitation services is an unavoidable priority.

### Policy stream

Several alternatives and policies can be adopted with regard to each function of financing (revenue collection, pooling of resources, purchasing, and benefit design) to improve PRF. These strategies would steer relevant decision- and policy-makers to strengthen PRF in Iran.

#### Sustainable financial resources

The interviewees expressed that resources such as direct and indirect taxes could provide sustainable financial resources for the rehabilitation services. A number of the participants suggested that the chemical industry, as well as oil and petrochemical companies, should pay special taxes to finance chronic diseases’ interventions, including rehabilitation. Receiving insurance premiums from cooperatives, which encompass many informal forces, was another proposed solution. In addition, allocating more premiums for childless families, as these individuals need more support services if they get disabled, was another solution.

#### External revenue sources

Annually, significant financial resources are allocated by international organizations such as the World Bank, the United Nations, and the WHO to provide optimal health and rehabilitation services to individuals with disabilities and chronic diseases worldwide. A number of policy-makers suggested that such resources should be used with a strong health diplomacy.

#### Earmarking the allocated resources

During the interview sessions, the participants stated that despite allocating funds to some rehabilitation services, these allocated resources are directed to other health sectors. In response, an insurance expert proposed that some mechanisms should be considered to ensure that the allocated resources are earmarked.

#### Integration of the current funds to have better pooling

As it was mentioned, the existence of various insurance funds leads to inappropriate pooling; hence, a number of the participants suggested that at least some funds should be integrated and merged to have effective national redistribution mechanisms.

### Incentive and timely payment mechanisms

A number of providers referred to poor and delayed repayments by insurance companies. They argued that such behaviors made many service providers reluctant to cooperate with insurers. Accordingly, strategies such as performance-based payments were proposed to increase the practitioners’ satisfaction.

#### Strategic purchasing principles

The respondents, pointing to the existing passive purchasing principles, suggested that health insurers must use strategic purchasing principles to enhance equity and minimize the potential corruption. Considering preventive and cost-effective interventions for inclusion in the basic health benefits package was the expressed solution regarding with issue.

#### Rationing strategies

Clear prioritization and rationing in accordance with scientific evidence was another solution proposed in this study by many university professors and policy-makers. In general, effective institutional and individual rationing not only controls staggering costs but also ensures access to basic services for the groups in need. In accordance with the development of scientific evidence and clinical guidelines in recent years, appropriate grounds were provided to consider PRS through rationing and prioritization.

#### Establishing new funds to cover long-term rehabilitation services

As a notable finding, a number of policy-makers stated that a new insurance fund, mainly financed by the government and public resources, can be established due to the cost of rehabilitation services for chronic diseases such as stroke. Also, using personal medical accounts to render improved risk-sharing over time can be another policy.

#### Increasing the proportion of rehabilitation services from government resources

In this study, some of the participants pointed to the need to increase the proportion of PRS in the government’s general budget. According to Article 78 of the Sixth Five-year Development Plan, OOP payments must be < 25% by the middle of 2021. Accordingly, these services, which have a high OOP rate, should be considered to achieve the concerned goal.

#### Using the capacity of supplementary insurances and NGOs

Some participants, especially university professors and policy-makers, highlighted the need to use the capacity of supplementary insurance and NGOs such as the Iranian Red Crescent Society and professional associations to improve PRF.

### Political stream

#### Parliament support

The participants argued that parliament always enacted appropriate laws on rehabilitation services and funding approaches; however, they did not receive much attention in the implementation process. For example, according to Article 89 of the Fourth 5-year Development Plan (F5DP), although the Ministry of Health is obligated to improve the referral system and prioritize the services, rehabilitation services are not considered part of health services in practice. Additionally, according to Article 92 of the F5DP, 10% of the price of third-party insurances must be credited to the MHME’s revenue account to be used to treat and rehabilitate the injured from traffic accidents. Other parliamentary supports are the enactment of Comprehensive Law on Protection of the Rights of Persons with disabilities (2004) and Law on Protection of the Rights of Persons with Disabilities (2018), which explicitly oblige the government to finance rehabilitation services by insurance mechanisms.

#### Changes in administrations

The study participants believed that the new government (President Rouhani’s government) has paid more attention to the health and welfare sector as well as its funding. In this regard, Rouhani promised a budget of 10,000 billion Rials to enforce the Law on Protection of the Rights of Persons with Disabilities. Accordingly, it is possible to take advantage of this situation to better cover PRS by basic insurances. On the other hand, the development of tax collection structures in recent years has provided the grounds for receiving premiums in a fair manner for PRF.

#### International effects

The participants noted that the introduction of “Rehabilitation 2030: A call for Action”, as well as “Rehabilitation in Health Systems” by WHO following the SDGs, attracted the academics and policy-makers’ attention to the field of rehabilitation. Moreover, the movement towards UHC, which includes rehabilitation services, also made policy-makers collaborate to provide the desired services in Iran.

#### Pressures from interest campaigns and NGOs

Many participants pointed to the creation and development of campaigns and NGOs in support of persons with disabilities after the formation of the Reformist Government in Iran, which progressed rapidly with the initiation of President Rouhani’s government and the improvement of the economic environment. Nowadays, the development of virtual networks has also enabled these actors to put pressure on the governing institutions.

#### International sanctions

Although after years of negotiations, the Joint Comprehensive Plan of Action (JCPOA) was signed in 2015, with the withdrawal of the United States, international sanctions were re-imposed in 2018. According to the findings, many participants believed that imposing sanctions and reducing Iran’s economic growth could also challenge PRF. Accordingly, the use of diplomatic approaches may help reduce the negative effects of these sanctions.

## Discussion

To narrow the existing gap between the demand for PRS and their supply, PRF must be taken into consideration. This qualitative study was conducted to assess policies and issues related to PRF financing in Iran. Kingdon’s multiple streams model was applied as an analytical framework to analyze the data gathered through political documents and semi-structured interviews. A host of factors of various natures were identified that impeded favorable PRF. PRS were under-prioritized by some policy-makers. The political climate of Iran was, however, recognized appropriate for PRF to be put on the national health political agenda. The role of NGOs and campaigns of people with disabilities to attract the policy-makers’ attention to the issue was emphasized.

In the problem stream, the problem should first be recognized. Different dimensions of the problem were illustrated by the interviewees. The systematic indicators delineated the magnitude and extent of the problem. Relative high cost of PRS, insufficient financial resources, inadequate and inefficient budget allocation, weak insurance coverage, and the increasing prevalence of cases requiring PRS were considered as the evident indicators of the problem. Given the growing demand for PRS [27], on the one hand, and the fact that financial issues are currently hindering equitable access to these services for those who need them [14, 25], the attention of politicians and policy-makers should be captured to adopt some appropriate measures promptly. A major issue inferred from some stakeholder interviews, including policy-makers and insurers, was the fact that they were unaware of the role and position of PRS in the health system as such they considered it insignificant and expensive compared to the other health services. Although physical rehabilitation needs per capita are increasing globally [36], it is widely recognized that the need for these services is being underestimated [37]. This implies that the feedback from stakeholders such as rehabilitation faculties, scientific associations, rehabilitation experts, and involved NGOs is inadequate to present the problem to policy-makers and politicians. This finding is consistent with Soltani et al.’s study [26]. In other words, feedback from the functioning system has not been strong enough to attract the policy-makers’ attention to the problems. This, on the one hand, results in a small share of the limited available financial resources to be allocated to this sector, and on the other hand, this small share would be used inefficiently. The aforementioned events and symbols are important factors in the problem stream and can forcefully present the problem and catch the policy-makers’ attention [33]. Iraq invasion to Iran in 1980, as a crisis, left many persons with disabilities in need of medical care, including PRS [38]. War invalids in many cases were conspicuously evocative of the PRS hardship and acted as symbols in the problem stream. These factors called for the establishment of support organizations, including the Martyr and Veterans Affairs Foundation, as well as the adoption of supportive policies. This is an example of what Kingdon considers to be the convergence of streams and a short-lived opportunity of a policy window opening [32]. Although these factors were important strides in attracting policy-makers’ attention to PRS, they were time-dependent and included certain groups of service recipients [27].

In the policy stream, potential solutions to the concerned problem are investigated to generate a policy or program proposal. The participants proposed a number of strategies regarding the different functions of PRF. According to Kingdon, a viable proposal should be technically feasible [31]. Reduction in oil revenue [39], economic recession, and international sanctions [40, 41] against Iran hinder approaches focusing on new internal or external revenue sources to find their ways to the political agenda. The policy alternatives, which emphasize the more effective and efficient allocation of resources and take advantage of the existing capacities, have higher likelihood of viability and credibility to be brought forth to the agenda-setting process [25]. PRS comprise a wide range of services dealing with a variety of health conditions and service beneficiaries. In this regard, some interviewees discussed strategies such as setting scientific prioritization matrices and rationing based on clinical practice guidelines to reduce the risk of deprivation of the ones in need of basic services and increase the access of the ones with more demanding conditions. The diversity of PRS, on the other hand, represents the involvement of various health care professionals in the field of physical rehabilitation [42, 43]. These disciplines include but are not limited to physical medicine and rehabilitation, physical therapy, occupational therapy, prosthetics and orthotics. The disagreements and conflicts of interest among these disciplines may lead to a lack of consensus, which in turn has a destructive effect on their potential role as influential policy communities outside the government [25]. The campaigns and NGOs of persons with disabilities were considered to be more influential by the interviewees, especially in recent years, and the role of virtual networks to represent national mood was also highlighted. No policy entrepreneur was detected in the analysis of the collected data in the policy stream. Given the significant role of policy entrepreneurs in policy development and introducing them to policy-makers [44, 45], the convergence of the streams is more challenging and less purposeful.

According to the participants, the political climate and events in the government have been appropriate for PRF to be raised on the national agenda. Legislatively, the upstream laws have paved the way for the politicians to consider the laws and regulations required for the enactment process. From the execution perspective, however, the enforcement of the existing PRF laws has not been much strict [27]. The current political context of Iran needs to be re-attracted to the issue of PRF. Although the political will seems to exist to confront this problem, appropriate strategies and approaches should be reorganized. This is to a large scale due to the economic consequences of the international sanctions against Iran, as documented in another study [40]. Feasible policy proposals, which are in accordance with the socio-economic situation and accepted by active political forces, are required to open a policy window [30, 33]. The significance of the mechanisms of active purposive policy-making and the potential role of their stakeholders are re-highlighted.

This study suffers from a number of limitations. During the study period, some individuals, especially health policy-makers were reluctant to participate; therefore, despite the efforts of the research team, the included participants were not completely representative of the target community. This limitation along with the descriptive nature of the study reduces the generalizability of the findings. Furthermore, a cross-sectional design was used in this study, which might lessen the validity of the findings.

## Conclusion

The recently enacted laws indicate that PRF-related policies have already been on the national health political agenda. This study, however, detected a set of multifaceted problems regarding PRF, such as lack of sustainable financial resources, fragmented financing, lack of sufficient awareness among decision- and policy-makers, and low participation of relevant experts in policy-making processes. Declining oil revenues, the economic recession, and international sanctions against Iran necessitate a focus on policy alternatives that make more effective use of the existing resources. In the presence of political will, the crucial role of policy-making and active purposive advocacy mechanisms are highlighted to open a window of opportunity and stimulate the agenda-setting process in line with UHC.

## Data Availability

The data collected and analyzed during the study are available from the corresponding author on reasonable request.

## Abbreviations

PRF: Physical rehabilitation financing
UHC: Universal Health Coverage
SDGs: Sustainable Development Goals
NCDs: Non-communicable diseases
CHEs: Catastrophic health expenditures
OOP: Out-of-pocket
PRS: Physical rehabilitation services
SSO: Social Security Organization
MHME: Ministry of Health and Medical Education
IHIO: Iran Health Insurance Organization
EMRO: Eastern Mediterranean Region Office
IUMS: Iran University of Medical Sciences
JCPOA: Joint Comprehensive Plan of Action
